# Hospital Plans and Their Selection

**Published:** 1914-07-11

**Authors:** 


### Hospital Plans and their Selection.

We have received a letter from Mr. A. Saxon Snell in reference to the article under this head in our issue of June 27, in which he states: "I do not believe that any responsible assessor ever made such conditions as that indicated [*i.e.* that any set of plans selected shall be adopted and carried out by the hospital authorities]. I know that the R.I.B.A. certainly never advise or countenance so obviously absurd an attitude. The most we stipulate for is that if the building owner does not adopt the assessor's award, adequate compensation (generally assessed at 1¼ per cent. upon the cost of the design) shall be paid to the author of the design. Under all the circumstances that is a reasonable condition."

